# Appointment in the Army

**Published:** 1862-01

**Authors:** 


### APPOINTMENT IN THE ARMY.

Dr. Levi J. Ham, of South Bend, Indiana, formerly of Williamsville, Erie County, N. Y., has been appointed Surgeon to the 48th Indiana Regiment, Col. Eddy, and has accepted his charge in Camp Ellis, Goshen. We most heartily congratulate the 48th Indiana Regiment upon this appointment.

